# Editorial: The scourge of zoonotic and veterinary important tapeworms

**DOI:** 10.3389/fvets.2025.1679061

**Published:** 2025-08-29

**Authors:** John A. Ohiolei, Erastus Mulinge

**Affiliations:** ^1^School of Molecular and Life Sciences, Curtin University, Bentley, WA, Australia; ^2^Kenya Medical Research Institute (KEMRI), Nairobi, Kenya

**Keywords:** *Echinococcus*, genetic diversity, DNA vaccines, cestodes, epidemiology, combination therapy

Tapeworms are ubiquitous and cause infections such as echinococcosis (*Echinococcus* spp.), cysticercosis and taeniasis (*Taenia* spp.), diphyllobothriasis (*Diphyllobothrium* spp.), Sparganosis (*Spirometra* spp.), and dipylidiasis (*Dipylidium caninum*) in humans and animals, resulting in significant morbidity, mortality, and substantial economic losses, particularly in resource-limited settings. The World Health Organization (WHO) has since recognized echinococcosis, taeniasis, and cysticercosis as Neglected Tropical Diseases (NTDs), and includes them among the 20 priority diseases in its *Roadmap for Neglected Tropical Diseases 2021–2030*, which aims for their control, elimination, or eradication by 2030 (1). Nonetheless, many tapeworm infections remain unprioritized and neglected (2). This Research Topic intends to highlight recent advances in tapeworm epidemiology and host-parasite interactions.

The epidemiology of tapeworms is shaped by a complex interplay of ecological and anthropogenic factors, particularly the presence and interaction of suitable intermediate and definitive hosts, including humans, dogs, livestock, fish, and wildlife. Effective control and management strategies often focus on disrupting the parasite's life cycle, either by preventing definitive hosts (e.g., canids in the case of cystic echinococcosis, CE) from seeding the environment with infective eggs or curbing indiscriminate disposal of infected offal that facilitates transmission to definitive hosts. In a large-scale livestock study by Abdelghani et al., CE prevalence up to 15%, was found with increased infection in older animals, likely due to repeated exposure over time. Similarly, Alene et al. investigated organ condemnation in slaughtered animals in Ethiopia, a country with one of Africa's largest livestock populations. They found that 7.5% of the animals had organs condemned due to infections with *E. granulosus* metacestodes or larval stage of *Taenia saginata*. Both studies underscore the persistent burden of tapeworm infections and highlight key epidemiological drivers, including host age, host species distribution, and husbandry practices. They also emphasize the need for integrated control strategies, such as genotyping of parasite populations, routine deworming, control of stray dogs, improved veterinary surveillance, and public education to discourage raw meat consumption.

Recent genomic and transcriptomic advances have significantly increased our understanding of the molecular mechanisms underlying host–parasite interactions, co-evolution, disease transmission dynamics, and the emergence of drug resistance (3). In tapeworms, particularly within the genus *Echinococcus*, considerable genetic diversity has been observed, often correlating with intermediate host distribution (4). In the study by Abdelghani et al., *Echinococcus granulosus* sensu stricto (G1 genotype), the most widespread and zoonotic variant, was yet identified as the predominant genotype in Tunisia. This genotype is especially common in sheep and has been reported on nearly every continent. The G1 genotype is also the primary target of the Eg95 vaccine, developed to prevent infection in intermediate hosts. However, studies have shown that orthologs of the Eg95 antigen vary in amino acid sequence across different genotypes, hence, the poor immunogenicity and efficacy in infection caused by non-G1 genotypes (5). Interestingly, no vaccines are commercially available to protect definitive hosts, even though they play a central role in the transmission of tapeworm infections. Although promising efforts are underway to develop vaccines against *Echinococcus* infection in definitive hosts [e.g., (6, 7)], progress remains constrained by poor understanding of the immunological mechanisms underlying parasite establishment and persistence (7).

In our Research Topic, two studies made attempts to address these problems. Firstly, Wang Z. et al., using RNA-sequencing analysis of transcription patterns observed in canine small intestinal epithelial cells, identified several differentially expressed genes involved in innate immune recognition, particularly those encoding pattern recognition receptors (PRRs) that detect pathogen-associated molecular patterns (PAMPs). PRRs play a vital role in initiating immune signaling cascades that lead to the recognition, containment, and clearance of invading pathogens. These genes represent valuable targets for identifying candidate molecules that may serve as biomarkers for diagnostics or as leads in drug and vaccine development (8). Next, the article by Wang N. et al., evaluated the immunoprotective potential of a DNA vaccine encoding the *E. granulosus* EgM123 protein in Beagle dogs. DNA vaccines have shown promise in combating various diseases, including COVID-19 and cancer, due to their capacity to induce strong cellular and humoral immune responses (9, 10). In their study, they found upregulation of key cytokines, including IL-1, IFN-γ, IL-4, and IL-6, along with marked levels of IgG. They observed an 87.85% reduction in worm burden and a 65.00 ± 15.52% inhibition in worm segment development, highlighting EgM123 as a promising vaccine candidate against *E. granulosus* infection in definitive hosts.

Besides CE, alveolar echinococcosis (AE), caused by *Echinococcus multilocularis*, poses unique clinical and therapeutic challenges. AE behaves similarly to a malignant tumor due to its infiltrative and metastatic-like growth, primarily targeting the liver and occasionally other organs. Management typically involves surgical resection, complemented by chemotherapy using anti-parasitic drugs. However, even after surgery, the parasite's ability to invade surrounding tissues and vasculature frequently leads to recurrence. Moreover, current treatments, like albendazole, have limited parasiticidal effect on *E. multilocularis* metacestodes and prolonged use (often the case) can cause significant toxicity (11). To address these limitations, studies have explored the use of combination therapy to enhance treatment efficacy, including improvements in drug bioavailability and synergistic antiparasitic effects (12, 13). Zhou et al., investigated the combined therapeutic effect of ubenimex, an immunomodulatory agent with anti-tumor properties, and albendazole in a BALB/c mouse model infected with *E. multilocularis* protoscoleces. Their findings showed that combination therapy significantly inhibited cyst growth and reduced cyst number, invasiveness, associated fibrosis, as well as reduced the levels of circulating biomarkers for liver damage compared to monotherapy. These results suggest that combination therapies targeting parasite survival and host immune modulation may offer new promise for AE management.

## Conclusion

The dynamic interactions between definitive hosts and intermediate hosts primarily drive the complex epidemiology of tapeworms. High prevalence rates of tapeworms in livestock have been consistently linked to factors such as host age, poor management of infected offal, and limited access to regular deworming programs and effective treatment options. Although we received a limited number of submissions, particularly those addressing other important tapeworm species, the studies included in our compilation highlight promising developments, such as the potential of combination therapies and DNA-based vaccines for definitive hosts, but significant gaps remain. Translating these scientific advances into practical and field-level interventions remains the critical turning point in achieving sustainable reductions in tapeworm burden.
